# Epidemiological, genetic, and clinical characterization by age of newly diagnosed acute myeloid leukemia based on an academic population-based registry study (AMLSG BiO)

**DOI:** 10.1007/s00277-017-3150-3

**Published:** 2017-10-31

**Authors:** Gabriele Nagel, D. Weber, E. Fromm, S. Erhardt, M. Lübbert, W. Fiedler, T. Kindler, J. Krauter, P. Brossart, A. Kündgen, H. R. Salih, J. Westermann, G. Wulf, B. Hertenstein, M. Wattad, K. Götze, D. Kraemer, T. Heinicke, M. Girschikofsky, H.G. Derigs, H. A. Horst, C. Rudolph, M. Heuser, G. Göhring, V. Teleanu, L. Bullinger, F. Thol, V. I. Gaidzik, P. Paschka, K. Döhner, A. Ganser, Hartmut Döhner, R. F. Schlenk, German-Austrian AML Study Group (AMLSG)

**Affiliations:** 1 Institute of Epidemiology and Medical Biometry, Ulm University, Helmholtzstr. 22, 89081 Ulm, Germany; 2 Department of Internal Medicine III, University Hospital Ulm, Albert-Einstein-Allee 23, 89081 Ulm, Germany; 3 Department of Internal Medicine I, Faculty of Medicine, University Hospital Freiburg, Freiburg, Germany; 4 Department of Internal Medicine II, University Hospital Hamburg-Eppendorf, Hamburg, Germany; 5 Department of Internal Medicine III, University Medical Center Mainz, Mainz, Germany; 6 Department of Internal Medicine III, Hospital Braunschweig, Braunschweig, Germany; 7 Department of Internal Medicine III, University Hospital of Bonn, Bonn, Germany; 8 Department of Hematology, Oncology and Clinical Immunology, University Hospital of Düsseldorf, Düsseldorf, Germany; 9 Department of Internal Medicine II, University Hospital of Tübingen, Tübingen, Germany; 10 Department of Hematology, Oncology and Tumor Immunology, Charité – Campus Virchow Clinic, Berlin, Germany; 11 Department of Hematology and Oncology, University Hospital of Göttingen, Göttingen, Germany; 12 Department of Internal Medicine I, Hospital Bremen-Mitte, Bremen, Germany; 13 Department of Hematology and Oncology, Hospital Essen-Werden, Essen, Germany; 14 Department of Internal Medicine III, University Hospital Klinikum rechts der Isar, Munich, Germany; 15 Department of Oncology and Hematology, Hospital Oldenburg, Oldenburg, Germany; 16 Department of Hematology and Oncology, University Hospital of Magdeburg, Magdeburg, Germany; 17 Department of Hematology and Oncology, Hospital Elisabethinen Linz, Linz, Austria; 18 Department of Internal Medicine III, Hospital Frankfurt-Hoechst, Frankfurt, Germany; 19 Department of Internal Medicine II, University Hospital of Schleswig-Holstein, Kiel, Germany; 20 Department of Hematology, Hemostasis, Oncology, and Stem Cell Transplantation, Hannover Medical School, Hannover, Germany; 21 Institute of Human Genetics, Hannover Medical School, Hannover, Germany; 22 NCT Trial Center, National Center for Tumor Diseases, Heidelberg, Germany

**Keywords:** Epidemiology, Genetics, Older age, AML, Registry

## Abstract

We describe genetic and clinical characteristics of acute myeloid leukemia (AML) patients according to age from an academic population-based registry. Adult patients with newly diagnosed AML at 63 centers in Germany and Austria were followed within the AMLSG BiO registry (NCT01252485). Between January 1, 2012, and December 31, 2014, data of 3525 patients with AML (45% women) were collected. The median age was 65 years (range 18–94). The comparison of age-specific AML incidence rates with epidemiological cancer registries revealed excellent coverage in patients < 70 years old and good coverage up to the age of 80. The distribution according to the European LeukemiaNet (ELN) risk categorization from 2010 was 20% favorable, 31% intermediate-1, 28% intermediate-2, and 21% adverse. With increasing age, the relative but not the absolute prevalence of patients with ELN favorable and intermediate-1 risk (*p* < 0.001), with activating *FLT3* mutations (*p* < 0.001), with ECOG performance status < 2 (*p* < 0.001), and with HCT-CI comorbidity index < 3 (*p* < 0.001) decreased. Regarding treatment, obesity and favorable risk were associated with an intensive treatment, whereas adverse risk, higher age, and comorbidity index > 0 were associated with non-intensive treatment or best supportive care. The AMLSG BiO registry provides reliable population-based distributions of genetic, clinical, and treatment characteristics according to age.

**Electronic supplementary material:**

The online version of this article (10.1007/s00277-017-3150-3) contains supplementary material, which is available to authorized users.

## Introduction

Acute myeloid leukemia (AML) is the most frequent acute leukemia in adults with an incidence of 3 to 4 per 100,000 persons per year [1, 2]. The median age at diagnosis ranges from 66 to 71 years [1, 3, 4]. Of note, over the last years the incidence has remained stable in younger patients but significantly increased in patients aged over 75 years [3, 5]. AML is a genetically very heterogeneous disorder characterized by the accumulation of somatically acquired genetic changes in hematopoietic progenitor cells that alter normal mechanisms of self-renewal, proliferation, and differentiation [6]. Treatment approaches are influenced by various factors, including patient features such as age, comorbidities, body mass index (BMI), and performance status as well as disease characteristics whereby the genetic profile of the disease is the most important prognostic factor [3, 7–11].

Scientific and technical advances accelerate the development and application of molecular genetic testing in subjects with leukemia. Mutations in the genes such as nucleophosmin-1 (*NPM1*), FMS-related tyrosine kinase 3 (*FLT3*), and CCAAT/enhancer-binding protein alpha (*CEBPA*) in cytogenetically normal AML influence the prognosis of AML patients [12] and have entered clinical routine [11, 13]. Activating *FLT3* mutations including internal tandem duplication (ITD) as well as tyrosine kinase domain (TKD) mutations and point mutations in exone 12 of *NPM1* are reported as frequent mutations in AML in an average young trial population with reported incidences of 33 and 28%, respectively [8], whereas mutations in *CEBPA* are less frequent [12]. In 2010, the European LeukemiaNet (ELN) proposed a standardized reporting system integrating cytogenetic and molecular genetic data which recently has been updated [13]. In addition, the efficacy and availability of *FLT3* inhibitors mark the starting point beyond acute promyelocytic leukemia of genotype-directed therapy in AML. Recently, the multi-kinase inhibitor midostaurin has shown efficacy in a randomized phase III trial of patients with activating *FLT3* mutations [14].

The introduction of personalized medicine will pose new challenges with respect to transition into clinical routine and in the evaluation of new treatment approaches in small genetically defined subgroups [8, 15]. Most of our knowledge on leukemia has been derived from center- and cooperative group-based clinical data rather than population-based registries [4, 16, 17]. Due to this increasing heterogeneity, registries are a valuable data source to appraise the presenting clinical characteristics and treatment decision in AML patients [4]. There is evidence from population-based registries that the survival expectations of patients with AML have improved over the past decades [17, 18], whereby in older AML patients only little or no progress has been made [5, 19, 20]. This difference probably reflects the difference in disease profile and frequency as well as severity of comorbidities in older compared to younger patients [3, 9, 13]. However, our understanding of genetic heterogeneity according to patients’ demographic, clinical, and treatment characteristics is still incomplete [17, 18].

The objectives of our study were to investigate epidemiological, genetic, and clinical characteristics of patients participating in the German-Austrian AML Study Group registry study (AMLSG BiO; ClinicalTrials.gov Identifier: NCT01252485) from 2012 to 2014, to compare them with selected epidemiological cancer registry data and to analyze distributions of genetic, clinical, and treatment characteristics according to age.

## Material and methods

### Study population

The AMLSG BiO registry study was initiated in 2010, and activation of most sites was completed in 2011. We aimed to analyze epidemiological, genetic, and clinical features between 2012 and 2014, representing a period of 2 years with full recruitment. We intended to register all patients aged 18 years or older with newly diagnosed AML at all centers of the German-Austrian AML Study Group (AMLSG) within the AMLSG BiO registry study. Via a web-based system, participating AMLSG centers registered patients with newly diagnosed AML based on local bone marrow and peripheral blood assessment after written informed consent. In all patients, bone marrow and peripheral blood samples were sent overnight by courier service to the AMLSG reference laboratories for cytogenetic and molecular genetic analyses (University of Ulm, Hannover Medical School). The study was approved by the ethical review boards of all participating centers.

For the current analysis, 3521 AML cases diagnosed between January 1, 2012, and December 31, 2014, were identified in the AMLSG BiO registry study database. Data were collected on sex, age, date of diagnosis, Eastern Cooperative Oncology Group (ECOG) performance status [21], and comorbidities according to the Hematopoietic Cell Transplantation-Specific Comorbidity Index (HCT-CI) [22]. AML cases were classified according to the 2008 World Health Organization (WHO) proposal [23] and risk-stratified according to the 2010 ELN classification [11]. In 3213 (91%) patients, information on treatment strategy was available including intensive chemotherapy, non-intensive treatment (azacitidine, decitabine, low-dose cytarabine), and best supportive care (BSC).

### Cytogenetics and molecular genetics

Chromosome banding analysis (within 14 days) and molecular screening (within 48 h) were performed in the two AMLSG central laboratories in Hannover and Ulm. Karyotypes were described according to the International System for Human Cytogenetic Nomenclature [24]. Leukemia samples were analyzed for mutations in *FLT3* (ITDs, and tyrosine kinase domain [TKD] mutations at codons D835/I836), *NPM1*, and *CEBPA* (both monoallelic and biallelic) as previously described [12, 25, 26].

The molecular profile of the disease in conjunction with a recommendation of potential trial participation was documented in a web-based system, and results were communicated immediately via facsimile to the participating centers.

### Statistical methods

Age-standardized incidence rates (ASR) of AML for Germany (*N* = 3251) were calculated using German population data in the years 2012–2014 [27]. Based on the geographic distribution of the contributing centers (Supplemental Fig. 1) with no or only few centers in Mecklenburg-Pommerania, Brandenburg, Thuringia, Saxony, and Bavaria, the data from these regions were excluded from the denominator of the population data.

For the AML diagnosis (ICD-9: 205.0 or ICD-10 C92.0), the incidence rates by sex and 5-year age classes were provided by the following cancer registries: Saarland (2006–2010), Bavaria (2010–2012), and North Rhine-Westphalia (2010–2013). Based on the data, mean annual incidence rates were calculated. In addition, data on AML from the Surveillance, Epidemiology, and End Results (SEER) program in the USA was used [28].

Multinomial logistic regression was calculated to estimate odds ratios (OR) and the 95% confidence intervals (95% CI) for treatment strategy (reference: intensive chemotherapy). The covariates age class, sex, ECOG performance status, ELN risk category, HCT comorbidity index, and BMI (< 20, 20–24, > 25 kg/m^2^) were entered simultaneously as independent variables into the final model to determine their effects on treatment decision.

## Results

**Fig. 1:**
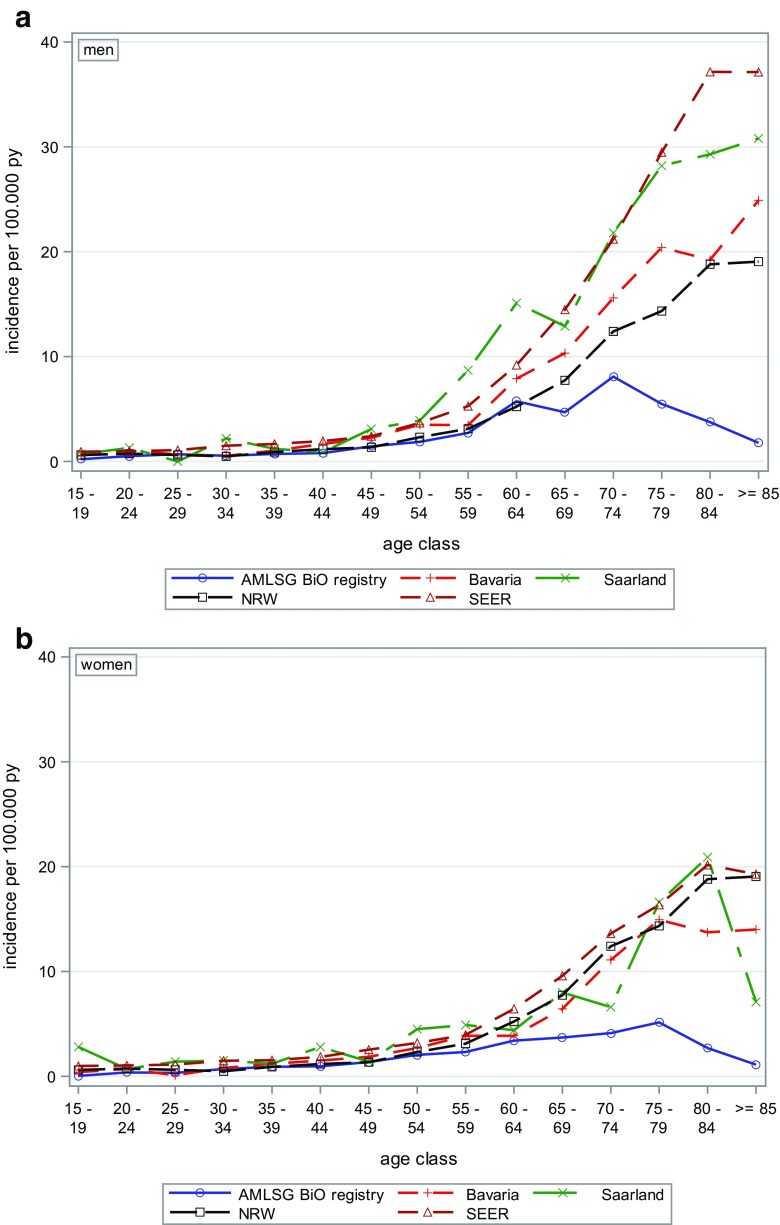
Incidence of AML 2012–2014 in the German AMLSG BiO registry (*N* = 3251) compared to selected German cancer registries and the US SEER program. AML in the cancer registries Bavaria, Saarland, and North Rhine-Westphalia (NRW) and US Surveillance, Epidemiology, and End Results (SEER) 2009–2013

Between 2012 and 2014, 3521 patients with newly diagnosed AML (45% women) were registered. The baseline characteristics of the AMLSG BiO study population are described in Table 1. Overall, the median age was 65 years (Q1, Q3; 54, 74, range 18–94); men were slightly older than women (median age 66 vs. 64 years). Independent of gender, most AML cases were observed in the age group 70 years and older (*N* = 1396, 39.7%). The age-specific incidence rates for AML in Germany are shown in Fig. 1a (for men), b (for women). The comparison of the age-specific AML incidence rates with data from German epidemiological cancer registries in Bavaria, North Rhine-Westphalia, and Saarland as well as the US SEER cancer database revealed good coverage of AML patients in the AMLSG BiO registry in younger and middle age classes, while older patients were less frequently registered. In both men and women, the incidence rates increased with age.

**Table 1:** Study population AMLSG BiO registry 2012–2014

		Total *N*=3,521	Men *N*=1,939	Women *N*=1,582	
		Median (Q1;Q3)	Median (Q1;Q3)	Median (Q1;Q3)	*p*-value*
Age (years)		65 (54;74)	66 (55;74)	64 (52;74)	0.0083
BMI (kg/m^2^)		26 (23;29)	26 (24;29)	25 (23;29)	<.0001
		N (%)	N (%)	N (%)	
Age classes (years)	<45	409 (11.62)	212 (51.83)	197 (48.17)	0.0091
	45 - 59	840 (23.86)	428 (50.95)	412 (49.05)	
	60 – 69	876 (24.88)	501 (57.19)	375 (42.81)	
	≥70	1,396 (39.65)	798 (57.16)	598 (42.84)	
Country	Germany	3,251 (92.33)	1,793 (55.15)	1,458 (44.85)	0.7322
	Austria	270 (7.67)	146 (54.07)	124 (45.93)	
Year of diagnosis	2012	994 (28.23)	556 (55.94)	438 (44.06)	0.7559
	2013	1,209 (34.34)	657 (54.34)	552 (45.66)	
	2014	1,318 (37.43)	726 (55.08)	592 (44.92)	
Type	de novo AML	2891 (81.1)	1580 (81.5)	1311(82.9)	<.0001
	secondary AML	478 (13.6)	295 (15.2)	183 (11.6)	
	therapy-related AML	152 (4.3)	64 (3.3)	88 (5.5)	
Prevalence of *FLT3*-ITD		666 (18.93)	306 (45.95)	360 (54.05)	<.0001
Prevalence of *FLT3*-TKD		219 (6.23)	109 (49.77)	110 (50.23)	0.1024
Prevalence of *NPM1* mutation		914 (25.97)	418 (45.73)	496 (54.27)	<.0001
Prevalence of *CEBPA*	monoallelic	86 (5.40)	40 (46.51)	46 (53.49)	0.0633
	biallelic mutation	70 (2.37)	43 (61.43)	27 (38.57)	
2010 ELN classification	Favorable	534 (20.18)	275 (51.50)	259 (48.50)	0.4590
	Intermediate-1	819 (30.95)	452 (55.19)	367 (44.81)	
	Intermediate-2	735 (27.78)	409 (55.65)	326 (44.35)	
	Adverse	558 (21.09)	308 (55.20)	250 (44.80)	
HCT-Comorbidity Index	0	1,337 (38.96)	715 (53.48)	622 (46.52)	0.1364
	1-2	1,152 (33.57)	639 (55.47)	513 (44.53)	
	≥ 3	943 (27.48)	544 (57.69)	399 (42.31)	
ECOG	0	1,416 (40.38)	777 (54.87)	639 (45.13)	0.7118
	1	1,491 (42.51)	809 (54.26)	682 (45.74)	
	2	452 (12.89)	262 (57.96)	190 (42.04)	
	3	129 (3.68)	73 (56.59)	56 (43.41)	
	4	19 (0.54)	11 (57.89)	8 (42.11)	
Study participation	Yes	591 (20.66)	299 (50.59)	292 (49.41)	0.0255
	No	2,270 (79.34)	1,265 (55.73)	1,005 (44.27)	
		N=3,213	N=1,760	N=1,453	
Therapy	Intensive	2,268 (71.12)	1,223 (53.92)	1,045 (46.08)	0.5646
	BSC	298 (9.34)	171 (57.38)	127 (42.62)	
	Non-intensive				
	AZA	113 (3.54)	60 (53.10)	53 (46.90)	
	DAC	243 (7.62)	142 (58.44)	101 (41.56)	
	LD AraC	267 (8.37)	148 (55.43)	119 (44.57)	

*AML* acute myeloid leukemia, *AZA* 5-azacytidine, *BMI* body mass index, *BSC* best supportive care, *CEBPA* CCAAT/enhancer-binding protein alpha, *DAC* decitabine, *ECOG* Eastern Cooperative Oncology Group performance status, *ELN* European LeukemiaNet, *FLT3* FMS-related tyrosine kinase 3, *HCT* hematopoietic cell transplantation, *ITD* internal tandem duplication, *LD AraC* low-dose arabinoside cytosine, *MDS* myelodysplastic syndrome, *N*, number of patients, *NPM1* nucleophosmin-1, *Q* quartile, *TKD* tyrosine kinase domain

^a^Chi^2^ test for categorical and Wilcoxon test for continuous variables

**Fig. 2:**
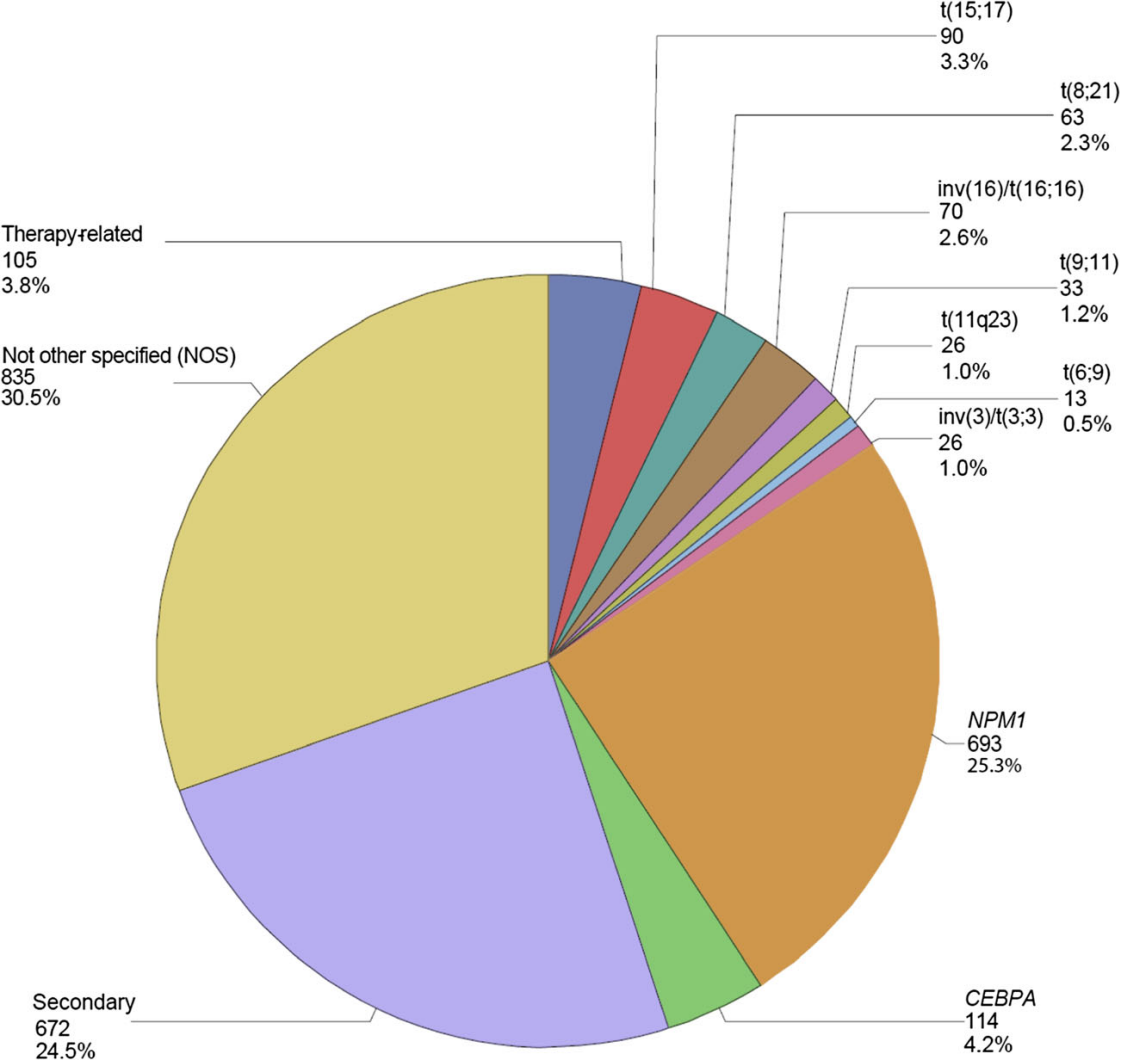
Distribution of AML subtypes (*N*, %) according to the WHO 2008 classification in 2740 patients. Abbreviations: *CEBPA* CCAAT/enhancer-binding protein alpha, *N* number of patients, *NPM1* nucleophosmin-1

In the AMLSG BiO registry, most registered cases were defined as de novo AML (82.1%), followed by secondary AML evolving after a myelodysplastic syndrome (13.6%) and therapy-related AML (4.3%). Mutated *NPM1* was present in 914 patients (26%), followed by *FLT3*-ITD in 666 (18.9%) and *FLT3*-TKD mutations in 219 (6.2%) cases. In women, *FLT3*-ITD and mutated *NPM1* were more prevalent than in men (both *p* values < 0.0001). According to the 2010 ELN classification, patients were diagnosed with favorable (*N* = 534, 20.2%), intermediate risk-1 (*N* = 819, 31%), intermediate risk-2 (*N* = 735, 27.8%), and adverse risk (*N* = 558, 21.1%). Categorization of cases according to the 2008 WHO classification revealed AML with recurrent genetic abnormalities as the largest subgroup (41.2%) including the two provisional entities AML with mutated *NPM1* (25.3%) and AML with mutated *CEBPA* (4.2%), followed by AML not otherwise specified (30.5%), AML with MDS-related changes (24.5%), and therapy-related AML (3.8%) (Fig. 2).

**Fig. 3:**
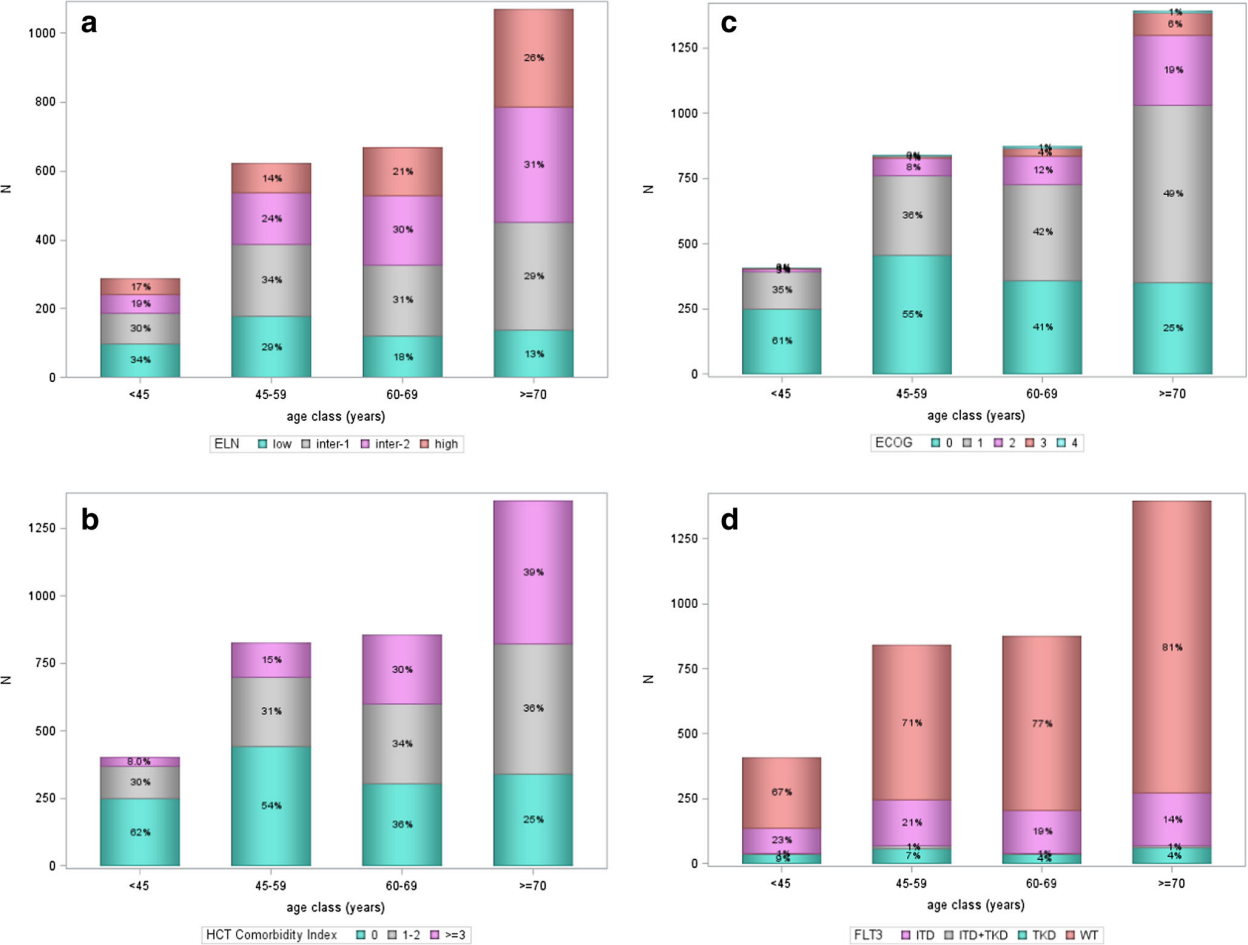
a–d 2010 European LeukemiaNet (ELN) classification, HCT comorbidity index, performance status (ECOG), and *FLT3* mutations by age classes in the AMLSG BiO registry (*N* = 3521). Abbreviations: *ELN* European LeukemiaNet, *ECOG* Eastern Cooperative Oncology Group performance status, *FLT3* FMS-related tyrosine kinase 3, *HCT* hematopoietic cell transplantation, *ITD* internal tandem duplication, *N* number of patients, *NPM1* nucleophosmin-1, *TKD* tyrosine kinase domain, *WT* wild type

Absolute and relative prevalence according to age groups as well as according to the 2010 ELN risk groups, HCT comorbidity index, ECOG performance status, and activating *FLT3* mutations are shown in Fig. 3 a–d. The figures show absolute numbers on the Y-axis and percentages in relation to the total number per age group in the bars. Overall, most patients were in the age group ≥ 70 years. The distribution of ELN risk groups changed significantly (*p* value < 0.001) with increasing age. In younger patients, the favorable risk group was either the most frequent (age < 45 years) or the second most frequent group (age 45–59 years), whereas in older patients (age 60–69 years, age > 70 years) the favorable risk group was the smallest subgroup. However, the absolute number of patients in the favorable risk group ranged between 65 and 192 patients and was comparable in all age groups (Fig. 3a). Thus, the relative prevalence of favorable-risk AML decreased whereas the absolute prevalence remained stable with increasing age. In contrast, the absolute and relative numbers of patients in the adverse risk group consistently increased with increasing age. Overall, most patients (39%) presented with no comorbidity burden (HCT-CI 0) with nearly stable absolute numbers across the different age groups, whereas the absolute and relative prevalence of patients with limited (HCT-CI 1–2) and extensive (HCT-CI ≥ 3) HCT comorbidity index increased with increasing age (Fig. 3b). A similar relationship was present for the ECOG performance status with a nearly stable absolute prevalence but decreasing relative frequency of fully active (ECOG 0) patients, whereas patients with slightly to moderately diminished performance status (ECOG 1/2) represented the largest group in older age groups (age 60–69 years, age ≥ 70 years) (Fig. 3c). Based on the performance status (ECOG ≤ 2), most of our patients were eligible for intensive chemotherapy even in the age group ≥ 70 years.

We were also interested in the distribution of patients with activating *FLT3*-ITD and *FLT3*-TKD mutations due to potentially available targeted therapy [14]. Although the relative numbers decreased significantly with increasing age, the absolute number of patients with activating *FLT3* mutations increased with age (< 45 years, *n* = 135; 45–59 years, *n* = 245; 60–69 years, *n* = 203; ≥ 70 years, *n* = 271; Fig. 3d). Based on reported very good response rates achieved in *NPM1*-mutated AML with intensive induction therapy [9–12], we performed an additional analysis according to *NPM1* mutational status and age. The percentages (absolute numbers) of patients with *NPM1* mutations according to the four age groups in ascending order were 24% (*n* = 100), 34% (*n* = 281), 27% (*n* = 238), and 21% (*n* = 295) indicating that even in age group ≥ 70 years a substantial number of patients could potentially benefit from intensive induction chemotherapy (suppl. Figure 3).

**Fig. 4:**
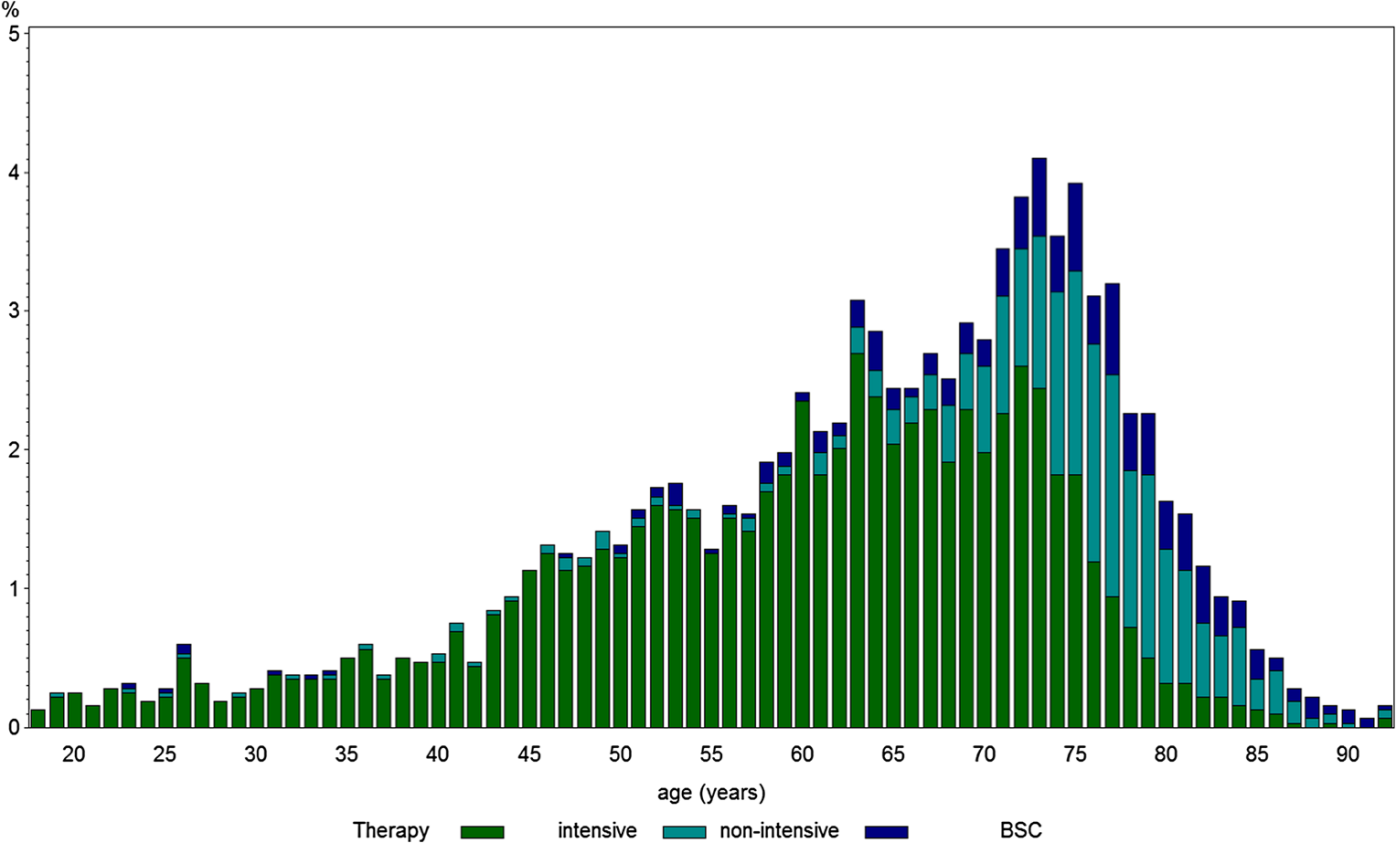
Frequency of treatment strategy (intensive, non-intensive and best supportive care (BSC)) according to age. Abbreviation: BSC, best supportive care ; N , number of patients

Most of the patients with information on treatment (*N* = 3213) received intensive chemotherapy (71.1%, Table 1). However, starting with the age of 65 years, a substantial and increasing proportion of patients received either non-intensive therapy or best supportive care (BSC) (Fig. 4) reaching a proportion of more than 50% in patients > 75 years.

In multivariate models (Table 2), age > 70 years was the strongest predictor to receive non-intensive treatment (OR, 9.91; 95% CI, 7.08–13.86; reference, intensive chemotherapy) or BSC (OR, 4.78; 95% CI, 3.17–7.22; reference, intensive chemotherapy), whereas younger age (< 60 years) was inversely associated with intensive chemotherapy. Other predictors for non-intensive treatment or BSC were ELN adverse-risk disease, comorbidity with an HCT-CI index ≥ 3, and impaired performance status (ECOG 2–4). With increasing comorbidity index, the association with non-intensive treatment (HCT-CI 1–2 vs. 0: OR, 1.30; 95% CI, 0.96–1.77; HCT-CI ≥ 3 vs. 0: OR, 1.65; 95% CI, 1.20–2.65) and BSC (HCT-CI 1–2 vs. 0: OR, 1.66; 95% CI, 1.10–2.57; HCT-CI ≥ 3 vs. 0: OR, 2.61; 95% CI, 1.68–4.07, respectively) became stronger. Of note, after adjustment for the other covariates, ELN favorable risk was only rarely associated with BSC as treatment strategy (OR 0.39 95% CI, 0.21–0.71), whereas non-intensive chemotherapy was nearly as frequently chosen as intensive chemotherapy (OR 0.95 95% CI, 0.65–1.39). Compared to BMI 20–24 kg/m^2^, obesity was less frequently associated with non-intensive treatment (OR, 0.86; 95% CI, 0.67–1.12) and BSC (OR, 0.57; 95% CI, 0.40–0.80).

**Table 2:** Multinomial logistic regression for therapy, sex, age classes, 2010 ELN classification, ECOG performance status, HCT comorbidity index, and BMI

Covariate		Intensive therapy (reference) (*N* = 1654)	
		Non-intensive^a^ (*N* = 460) Odds ratio (95% CI)	BSC (*N* = 207) Odds ratio (95% CI)
Age class (years)	< 60	0.51 (0.31, 0.84)	0.76 (0.43, 1.34)
	60–69	1	1
	≥ 70	9.91 (7.08, 13.86)	4.78 (3.17, 7.22)
Sex	Woman	0.97 (0.76, 1.24)	0.89 (0.64, 1.23)
	Men	1	1
ELN 2010 classification	Favorable	0.95 (0.65, 1.39)	0.39 (0.21, 0.71)
	Intermediate-1	1	1
	Intermediate-2	1.10 (0.81, 1.51)	1.13 (0.76, 1.67)
	Adverse	1.60 (1.16, 2.23)	1.17 (0.76, 1.79)
HCT comorbidity index	0	1	1
	1–2	1.30 (0.96, 1.77)	1.66 (1.10, 2.57)
	≥ 3	1.65 (1.20, 2.26)	2.61 (1.68, 4.07)
ECOG	0–1	1	1
	2–4	1.94 (1.42, 2.65)	4.31 (3.01, 6.16)
BMI (kg/m^2^)	<20	1.58 (0.91, 2.76)	1.46 (0.77, 2.78)
	20–24	1	1
	≥ 25	0.86 (0.67, 1.12)	0.57 (0.40, 0.80)

*BMI* body mass index, *BSC* best supportive care, *CI* confidence interval, *ECOG* Eastern Cooperative Oncology Group performance status, *ELN* European LeukemiaNet, *HCT* hematopoietic cell transplantation, *N* number of patients

^a^Including azacitidine, decitabine, and low-dose cytarabine

## Discussion

Based on the absolute numbers of 1307 patients included in 2014, the academic population-based AMLSG BiO registry study roughly represents one third of expected cases per year with newly diagnosed AML in Germany and Austria with an assumed incidence rate of 4 of 100,000 inhabitants. However, compared to other population-based cancer registries, the age-specific incidence rate of the AMLSG BiO registry is lower for persons of older age. Compared to the data of other cancer registries, about 20% of men aged over 70 years and women aged over 75 years with AML were registered in the AMLSG BiO registry suggesting that older AML patients were underreported. Possible explanations are that older patients were less frequently referred to specialized leukemia-treating hospitals and that in older AML patients genetic information was considered less relevant for the treatment decision. However, median age of 65 years at diagnosis in our AMLSG BiO registry was consistent with data reported from the Netherlands with a comparable population [17]. Thus, data from our AMLSG BiO registry shows an excellent and good population representation up to the age of 70 years and with an age between 70 and 80 years, respectively.

In our population-based approach, the distribution of selected genetic markers such as activating *FLT3* mutations and mutated *NPM1* differed in a clinically relevant manner from that recently reported in a large group of patients, all of them treated in interventional clinical trials with a median age below 60 years [8]; interestingly, the occurrence of *NPM1* mutations was very similar whereas the number of activating *FLT3* mutations was lower by a factor of 1.32. This clearly reflects patient selection towards younger and fitter patients when treated in clinical trials. As illustrated in Fig. 3d with a nearly stable absolute prevalence of patients exhibiting activating *FLT3* mutations in all age groups, the relative prevalence significantly decreased from 33, 29, 24, to 19% in the age groups < 45, 45–59, 60–69, and ≥ 70 years. This observation is consistent with findings showing that the frequency of *FLT3*-ITD and *FLT3*-TKD in adults decreases with increasing age [29]. However, with regard to the public health perspective and the planning of future clinical trials with FLT3 inhibitors, our results provide a good data basis in terms of expected absolute and relative numbers in the different age groups showing that the absolute number of older patients with activating *FLT3* mutations (≥ 60 years) still exceeds that observed in younger patients (< 60 years).

In the AMLSG BiO registry, most patients with AML are older with a high proportion of patients with adverse genetics (Fig. 3a), presence of comorbidities (Fig. 3b), and impaired performance status (Fig. 3c). However, up to the age of 75 years, most patients receive intensive chemotherapy (Fig. 4), while beyond this age more patients are treated with hypomethylating agents, low-dose cytarabine, or best supportive care. Consistent with the literature, in our study, impaired performance status and increased comorbidity index were more frequent in older age [30]. There has been a discussion about the cofactors influencing the treatment decision such as performance status and the burden of comorbidities [3] as well as the genetic profile of the disease [9]. Due to all these factors, the treatment of AML in older patients remains quite challenging [30], since intensive chemotherapy is toxic and less well tolerated by the older AML patients. Recent developments of targeted treatment for AML patients with activating *FLT3* mutation have drawn the attention to this subgroup, which comprises about one quarter of our registry patients. Although the relative number of activating *FLT3* mutation decreases with increasing age, still a substantial proportion of patients above the age of 60 years may benefit from specific *FLT3* inhibitors [31]. Thus, the differences of the genetic and clinical profiles between older and younger AML patients have to be taken into account when treatment strategies are developed [32]. A substantial proportion of older AML patients exhibit a *NPM1* mutation, which predicts for high response rates to intensive induction therapy even in the older patients with complete remission (CR) rates as high as 80% [33, 34]. This genetic marker information may therefore be used to guide treatment strategy in older patients.

After adjusting for disease and performance (ECOG, HCT-CI) parameters, we found overweight to be associated with the intensive treatment approach rather than non-intensive treatment and BSC. This suggests that obese patients are considered to tolerate intensive chemotherapy better compared to normal or underweight patients. This observation is paralleled by the finding that obese older AML patients have a better survival [35]. Whether the higher treatment intensity or other factors are responsible for superior survival of obese older patients remains elusive.

In conclusion, our study characterizes the academic population-based AMLSG BiO registry, which has excellent and good population coverage up to the age of 70 years and between 70 and 80 years, respectively. Our study indicates that the distribution of the genetic profile differs in a clinically fashion relevant by age. Taking into account relative and absolute numbers by age group, our study provides valid data for public health evaluations and the planning of interventional studies in genetically defined subgroups.

## Electronic supplementary material

ESM 1 (DOCX 546 kb)
