# Supplementary material for: Epidemiological, genetic, and clinical characterization by age of newly diagnosed acute myeloid leukemia based on an academic population-based registry study (AMLSG BiO)
Source: Ann Hematol. 2017 Oct 31;96(12):1993–2003. doi: 10.1007/s00277-017-3150-3 (PMC5691091; doi:10.1007/s00277-017-3150-3)
Supplement: Supplementary file 1 — (DOCX 546 kb) [file 277_2017_3150_MOESM1_ESM.docx]

**Supplemental Material to**

**Epidemiological, genetic and clinical characterization of newly diagnosed acute myeloid leukemia based on an academic population-based registry study (AMLSG BiO)**

Nagel G^1^, Weber D^2^, Fromm E^1^, Erhardt S^1^, Lübbert M^3^, Fiedler W^4^, Kindler T^5^, Krauter J^6^, Brossart P^7^, Kündgen A^8^, Salih HR^9^, Westermann J^10^, Wulf G^11^, Hertenstein B^12^, Wattad M^13^, Götze K^14^, Kraemer D^15^, Fischer T^16^, Girschikofsky M^17^, Derigs HG^18^, Horst HA^19^, Rudolph C^2^, Heuser M^20^, Göhring G^21^, Teleanu V^2^, Bullinger L^2^, Thol F^20^, Gaidzik VI^2^, Paschka P^2^, Döhner K^2^, Ganser A^20^, Döhner H^2^, Schlenk RF^2,22^, German-Austrian AML Study Group (AMLSG)

**Supplemental Figure 1: Recruitment in the AMLSG BiO registry in Austria (N= 270)***


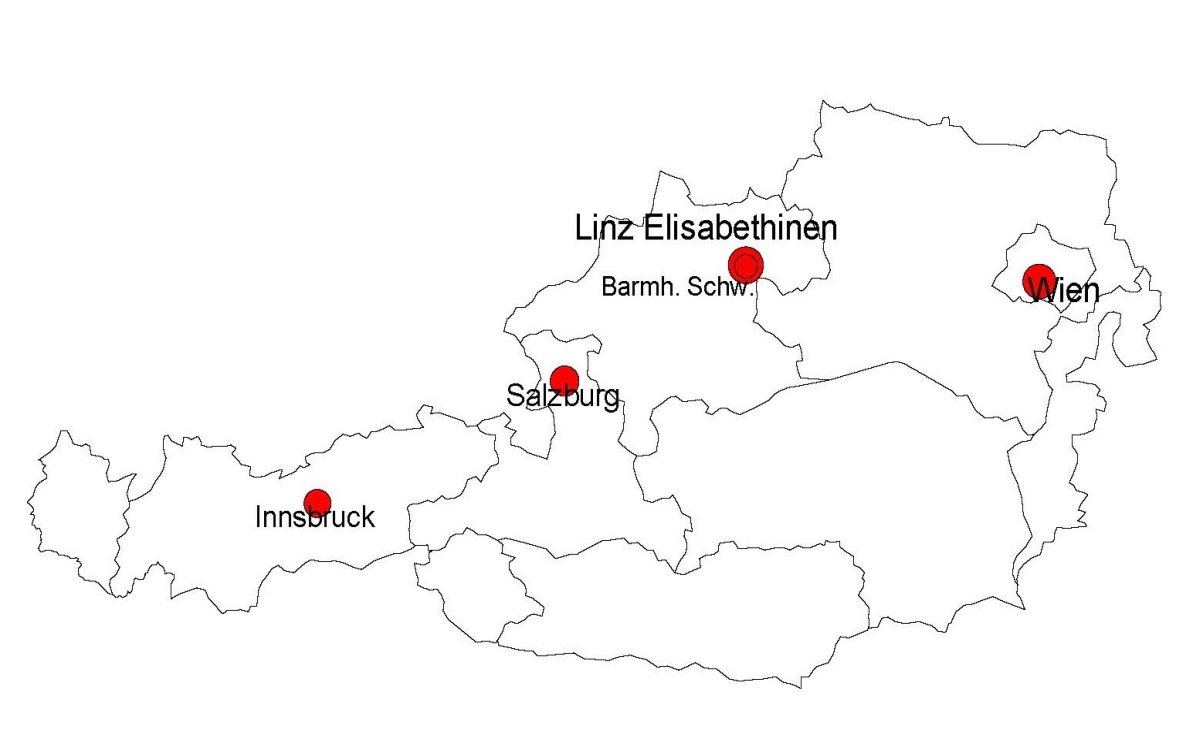


*Size of the dots corresponds to the number of reported cases of AML

**Supplemental Figure 2: Recruitment in the AMLSG BiO registry in Germany (N=3,251)**


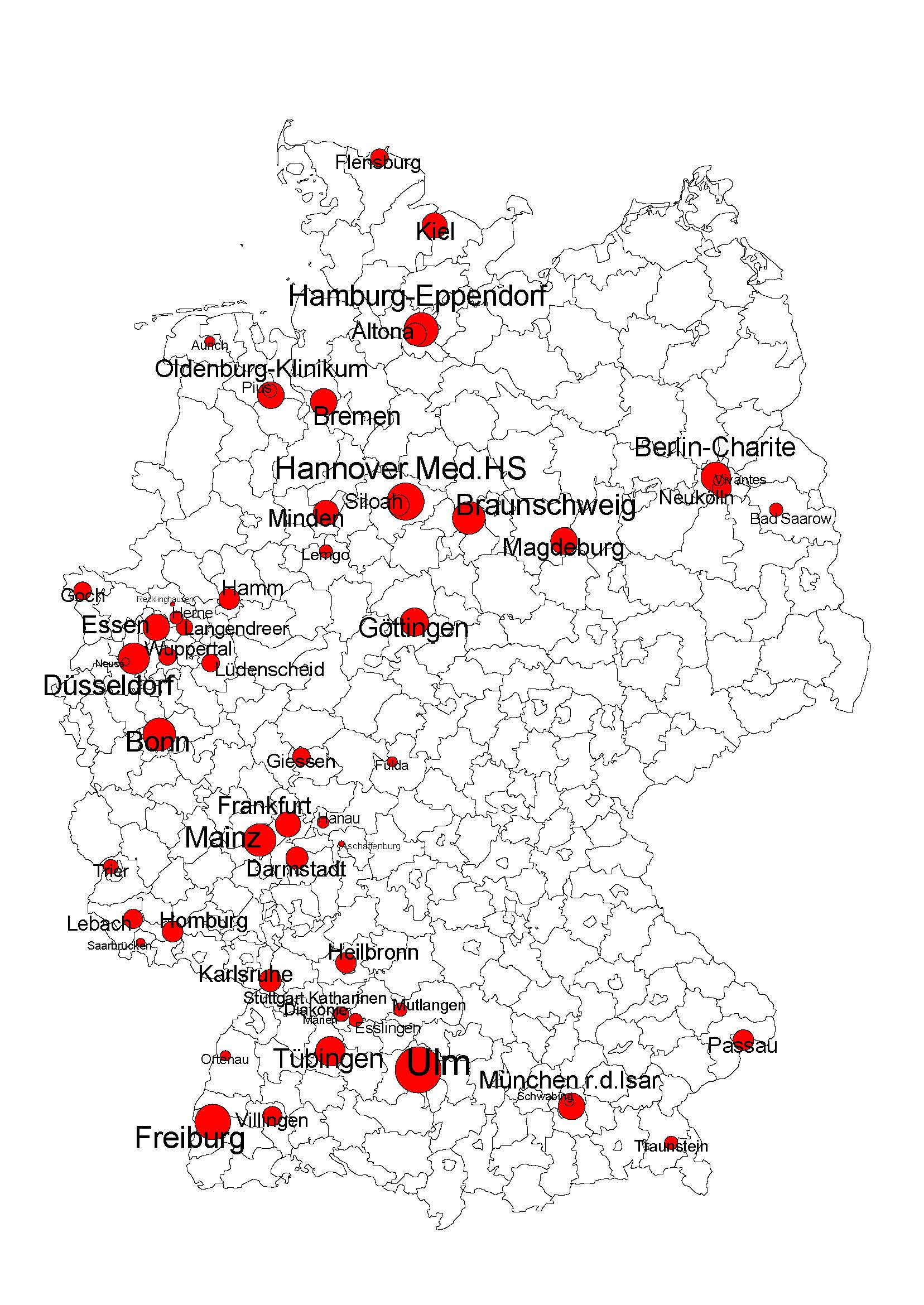


*Size of the dots corresponds to the number of reported cases of AML

| **Supplemental Table 1: Study population AMLSG BiO registry 2012-2014 in the multivariate model** | | | | | |
| --- | --- | --- | --- | --- | --- |
| **Covariate** | | | **Total**  **N=2,336** | **Men**  **N=1,273** | **Women**  **N=1,063** |
|  | | | **Median (Q1;Q3)** | **Median (Q1;Q3)** | **Median (Q1;Q3)** |
| **Age (years)** | | | 66 (55;74) | 68 (57;74) | 65 (53;74) |
| **BMI (kg/m^2^)** | | | 26 (23;29) | 26 (24;29) | 25 (23;29) |
|  | | | **N (%)** | **N (%)** | **N (%)** |
| **Age classes (years)** | < 59 | | 770 (32.96) | 372 (48.31) | 398 (51.69) |
| 60 - 69 | | | 608 (26.03) | 349 (57.40) | 259 (42.60) |
| ≥70 | | | 958 (41.01) | 552 (57.62) | 406 (42.38) |
| **Prevalence of *FLT3-*ITD** | | | 457 (19.56) | 200 (43.76) | 257 (56.24) |
| **Prevalence of *FLT3-*TKD mutation** | | | 138 (5.91) | 72 (52.17) | 66 (47.83) |
| **Prevalence of  *NPM1* mutation** | | | 650 (27.83) | 287 (44.15) | 363 (55.85) |
| **Prevalence of *CEBPA* mutation** | | | 136 (5.83) | 70 (51.47) | 66 (48.53) |
| **2010 ELN classification** | | Favorable | 479 (20.51) | 250 (52.19) | 229 (47.81) |
|  | | Intermediate-1 | 722 (30.91) | 393 (54.43) | 329 (45.57) |
|  | | Intermediate-2 | 652 (27.91) | 361 (55.37) | 291 (44.63) |
|  | | Adverse | 483 (20.68) | 269 (55.69) | 214 (44.31) |
| **HCT-Comorbidity index** | | 0 | 920 (39.38) | 480 (52.17) | 440 (47.83) |
|  | | 1-2 | 787 (33.69) | 425 (54.00) | 362 (46.00) |
|  | | ≥ 3 | 629 (26.93) | 368 (58.51) | 261 (41.49) |
| **ECOG** | | 0-1 | 1,957 (83.78) | 1,060 (54.16) | 897 (45.84) |
|  | | 2-4 | 379 (16.22) | 213 (56.20) | 166 (43.80) |
| **Therapy** | | Intensive | 1,663 (71.19) | 888 (53.40) | 775 (46.60) |
|  | | BSC | 211 (9.03) | 124 (58.77) | 87 (41.23) |
|  | | Non-intensive* | 462 (19.78) | 261 (56.49) | 201 (43.51) |
| Abbreviations: BMI, body mass index; BSC, best supportive care; ECOG, Eastern Cooperative Oncology Group performance status; ELN, European LeukemiaNet ; HCT, Hematopoietic cell transplantation; N, number of patients; Q, quartile.  *Including azacitidine, decitabine, and low-dose cytarabine | | | | | |

**Supplemental Figure 3: Frequency of *NPM1* mutation by age groups**
